# *De Novo* Genome Assembly of Stinkhorn Mushroom *Clathrus columnatus* (Basidiomycota, Fungi) Using Illumina and Nanopore Sequencing Data

**DOI:** 10.1128/mra.01026-21

**Published:** 2022-02-10

**Authors:** Eri Ogiso-Tanaka, Hiyori Itagaki, Muneyuki Ohmae, Tsuyoshi Hosoya, Kentaro Hosaka

**Affiliations:** a Center for Molecular Biodiversity Research, National Museum of Nature and Science, Tsukuba, Ibaraki, Japan; b Department of Biological Science, Graduate School of Science, The University of Tokyo, Bunkyo-ku, Tokyo, Japan; c Hokken Co. Ltd., Mibu-machi, Tochigi, Japan; d Department of Botany, National Museum of Nature and Science, Tsukuba, Ibaraki, Japan; University of California, Riverside

## Abstract

We report the reference genome of *Clathrus columnatus* isolate MO-923, which was isolated from Chichijima Island, the Ogasawara (Bonin) Islands, Japan. Oxford Nanopore Technologies MinION and Illumina sequence reads were assembled using NECAT and polished using Pilon to yield a 36.51-Mb genome with 10,625 predicted protein-coding genes.

## ANNOUNCEMENT

The basidiomycete *Clathrus columnatus* is a stinkhorn fungus in the family Clathraceae (subclass Phallomycetidae, order Phallales) (1). The species was originally described from South Carolina, USA, but was later reported from Africa, Australasia, Central America, and South America (2). In Japan, it was first collected from Hahajima, the Ogasawara (Bonin) Islands (3), but was later reported from wider areas, including both Hahajima and Chichijima Islands (4, 5).

The material for this study was collected at Mount Chuo, Chichijima Island (27°04′28.9″N, 142°13′07.1″E), on 7 November 2018. In the laboratory, immature fresh fruit bodies were dissected in half using a clean, sterilized razor blade, and a small piece of internal tissue was placed on potato dextrose agar (PDA) (Nissui, Japan) to obtain an isolate. After confirmation of mycelial growth without obvious contamination, the isolate was transferred to PDA slants and was maintained as isolate MO-923 until further experiments. The remaining material was photographed and dried for 24 h with low heat (∼45°C) and good air circulation using a food dehydrator. A dried voucher specimen was deposited at the fungal herbarium of the National Museum of Nature and Science (TNS) under registration number TNS-F-82131 for future studies.

Prior to DNA extraction, the isolate of *Clathrus columnatus* was transferred again to PDA plates prepared in 9-cm petri dishes. Ten inoculated PDA plates were incubated at 20°C in the dark. After 2 weeks, fragments of mycelial colonies were harvested by peeling them from the agar using sterilized tweezers. DNA was extracted by the method described at protocols.io (6).

The library preparation for the Illumina platform was performed using the NEBNext Ultra II FS DNA library preparation kit for Illumina (New England Biolabs, USA) following a modified manufacturer’s protocol (7). The library was then sequenced on an Illumina MiSeq instrument to obtain 300-bp paired-end reads using a 600-cycle v3 kit (Illumina, USA) (Table 1).

**TABLE 1:** Genome assembly, Nanopore sequencing, and Illumina sequencing data

Parameter	Finding
Sample	*Clathrus columnatus* isolate MO-923
No. of contigs	19
Total length (bp)	36,509,728
*N*_50_ (bp)	3,174,166
GC content (%)	41.3
Coverage (×)	164
Nanopore sequencing	
No. of reads	1,108,254
No. of bases	8,298,334,373
Avg length (bp)	7,488
Illumina sequencing	
Total no. of reads	4,585,114
Total size (Mb)	2,348

The genomic DNA (gDNA) (1 μL) was diluted in EB buffer to a final volume 100 μL and sheared in g-TUBEs (Covaris, USA) with two passages at 4,000 × *g* or 1,300 × *g* for 1 min in a centrifuge (TOMY, Japan). The library preparation was performed using the ligation sequencing kit SQK-LSK109 (Oxford Nanopore Technologies, United Kingdom) following the manufacturer’s protocol. The library was then sequenced on a MinION system using R9.4.1 flow cell sequencing chemistry with MinKNOW v4.2.5 software. Guppy GPU v4.5.2+bcc53d3 (Oxford Nanopore Technologies) was used for base calling (Table 1). For the long-read genome assembly, Filtlong v0.2.0 (https://github.com/rrwick/Filtlong) was used to extract the top 278,079 longer reads (total of 5 Gbp) and high-quality reads from the 1.1 million reads obtained (8.30 Gbp). The filtered Nanopore reads were assembled using NECAT (8). For Illumina short reads, quality control was performed with Trimmomatic v0.30 (9). Then, the processed reads were used for polishing of the sequences assembled from Nanopore reads. Six rounds of assembly polishing were performed with Pilon v1.13 (10) using Illumina reads aligned with BWA v0.7.9a (11) for short-read mapping. As a result, we obtained a complete genome assembly with 19 contigs containing 36,509,728 bp (GC content, 41.27%). The *N*_50_ value for the contigs was 3,174,166 bp, and the Benchmarking Universal Single-Copy Orthologs (BUSCO) completeness value was 92.07% (BUSCO v3 fungi_odb9 [12] and QUAST v5.0.2 [13]). Annotation of the assembled genome was carried out using the Funannotate pipeline v1.5.2 (14), which included masking repetitive elements with the RepeatMasker tool (https://www.repeatmasker.org) and annotating genes with AUGUSTUS (15), GlimmerHMM (16), SNAP (17), and EVM (18). Gene function was predicted using the Pfam (19), MEROPS (20), CAZy (21), InterProScan (22), and Swiss-Prot (23) databases. A total of 10,625 protein-coding genes were predicted.

### Data availability.

The annotated complete genome assembly of *Clathrus columnatus* isolate MO-923 is available in GenBank and DDBJ under the accession numbers GCA_020884735.1 and BPWL01000001 to BPWL01000019. A table with all Web addresses was uploaded at figshare (https://figshare.com/articles/online_resource/Data_availability_Clathrus_columnatus_genome/17246096). The Illumina MiSeq and Oxford Nanopore Technologies sequencing raw data were deposited in DDBJ under the accession numbers DRA012831 and DRA012832 (BioProject accession number PRJDB12304). The voucher specimen (dried specimen) from which the isolate was obtained is housed at the fungal herbarium of the Department of Botany, TNS, under the registration number TNS-F-82131.
